# Generative multi-adversarial network for striking the right balance in abdominal image segmentation

**DOI:** 10.1007/s11548-020-02254-4

**Published:** 2020-09-08

**Authors:** Mina Rezaei, Janne J. Näppi, Christoph Lippert, Christoph Meinel, Hiroyuki Yoshida

**Affiliations:** 1 Hasso Plattner Institute, Prof.Dr. Helmert Street 2-3, Potsdam, Germany; 2 Massachusetts General Hospital and Harvard Medical School, 25 New Chardon St., Boston, MS USA

**Keywords:** Imbalanced learning, Generative multi-discriminative networks, Semantic segmentation, Abdominal imaging

## Abstract

**Purpose** The identification of abnormalities that are relatively rare within otherwise normal anatomy is a major challenge for deep learning in the semantic segmentation of medical images. The small number of samples of the minority classes in the training data makes the learning of optimal classification challenging, while the more frequently occurring samples of the majority class hamper the generalization of the classification boundary between infrequently occurring target objects and classes. In this paper, we developed a novel generative multi-adversarial network, called Ensemble-GAN, for mitigating this class imbalance problem in the semantic segmentation of abdominal images.

**Method** The Ensemble-GAN framework is composed of a single-generator and a multi-discriminator variant for handling the class imbalance problem to provide a better generalization than existing approaches. The ensemble model aggregates the estimates of multiple models by training from different initializations and losses from various subsets of the training data. The single generator network analyzes the input image as a condition to predict a corresponding semantic segmentation image by use of feedback from the ensemble of discriminator networks. To evaluate the framework, we trained our framework on two public datasets, with different imbalance ratios and imaging modalities: the Chaos 2019 and the LiTS 2017.

**Result** In terms of the F1 score, the accuracies of the semantic segmentation of healthy spleen, liver, and left and right kidneys were 0.93, 0.96, 0.90 and 0.94, respectively. The overall F1 scores for simultaneous segmentation of the lesions and liver were 0.83 and 0.94, respectively.

**Conclusion** The proposed Ensemble-GAN framework demonstrated outstanding performance in the semantic segmentation of medical images in comparison with other approaches on popular abdominal imaging benchmarks. The Ensemble-GAN has the potential to segment abdominal images more accurately than human experts.

## Introduction

One of the major challenges of deep learning for medical image analysis is the highly skewed class distribution of objects in medical images, which is referred to as the imbalanced classification problem. An imbalanced classification problem occurs when the target classes of a dataset have a highly unequal number of samples. For example, in a binary classification, the imbalanced classification problem occurs when the number of samples representing a specific disease has fewer observations than the healthy class. The former is called an infrequent class or minority class, whereas the latter is called a majority class. Because canonical machine learning assumes that different categories have similar numbers of samples, a model trained on such imbalanced data distribution will be biased toward the most frequent class, which is not desirable in clinical applications.

In this work, we mitigate the negative impact of the class imbalance problem through ensemble learning of discriminative convolutional neural networks. By combining multiple networks that are individually complementary, one can obtain a compound classifier that is more accurate than any of its base components [1]. Here, we propose an architecture based on a generative multi-adversarial network, called Ensemble-GAN, which is composed of a generator and an ensemble of discriminators. We implemented the generator network in a multi-discriminator setting through simultaneous minimization of different losses to minimize the prediction error of the generator model as a multi-objective optimization problem. The discriminators were varied by use of different feature maps, different losses, and initializations. Moreover, we developed methods for providing more accurate semantic segmentation of high-resolution medical images than existing approaches.

To demonstrate the generalization ability of our approach, we evaluated the performance of the Ensemble-GAN in semantic segmentation of organs and tumor regions from abdominal computed tomography (CT) and magnetic resonance (MR) images by use of a highly imbalanced training dataset where the number of pixels belonging to abnormal regions of interest was much smaller than that of normal regions. The results demonstrated the generalization ability of our approach in the segmentation of body organs and tumor regions.

The rest of the paper is organized as follows: “Related work” section presents an overview of the most recent approaches to the imbalanced classification problem and semantic segmentation of medical images. “Methoda” section explains the proposed approach for learning the class imbalance problem. The experimental design and results are presented in “Experimental design” and “Results” sections, respectively, followed by the discussions and conclusions in “Discussions and conclusions” section.

## Related work

This section provides a brief review of the most recent state-of-the-art approaches carried out on the topics of learning from imbalanced data, multi-objective training of generative adversarial networks (GANs), and medical image segmentation.

### Learning from imbalanced data

In medical image analysis, the most popular strategies for addressing the imbalanced classification problem have included data-level methods and algorithmic methods. The data-level methods include under-sampling or over-sampling of the training dataset. However, these resampling approaches often remove some of the important samples or they add redundant samples to the training data. Algorithmic methods have included cost-sensitive learning and ensemble learning. The cost-sensitive learning is typically used with accuracy loss [2], Dice coefficient loss [3], and asymmetric similarity loss [4] to modify the distribution of the training data based on a mis-classification cost. However, in the case of image segmentation, losses such as mean surface distance or Hausdorff surface distance are more appropriate. Most of the imbalanced ensemble techniques apply majority voting [5] or average voting [6] with a combination of losses and different initializations. The trade-off from the bias and variance of combining a redundant ensemble was studied by Sun et al. [7]. Because the ensemble model reduced the variance on test data, the prediction result for the minority class was improved [7].

### Multi-objective training of GANs

Recently, variants of GAN models have included multiple generators and/or multiple discriminators to tackle the problems of mode collapse, global optimization, and non-convergence of conventional GANs. Durugkar et al. [8] introduced a generator with multiple discriminators, where the average or maximum of discriminator losses provides feedback to the generator. In another study, a generator was trained with a set of discriminators where each discriminator classified a fixed random projection of the inputs [6, 9]. In contrast, the MGAN [10] and MAD-GAN [11] schemes proposed GAN-based architectures with multiple generators and single discriminator, while the MD-GAN [12] introduced a distributed GAN composed of four generators and four discriminators. Sathish et al. [13] (IITKGP-KLIV) performed adversarial learning composed of two auxiliary classifiers and one discriminator with application to medical image segmentation.

### Semantic image segmentation

Recent studies on deep learning for semantic segmentation of images have differed mostly in terms of their architectural design for linking different parts of the image to reveal relationships between the objects. Examples include the DeepLabv3+ [14] framework which used an encoder–decoder structure with the separable atrous convolution composed of a depth-wise convolution (spatial convolution for each channel of the input) and point-wise convolution (1$\times$1 convolution with the depth-wise convolution as input). Pham et al. [13] (ISUDE) proposed an hourglass autoencoder with DICE loss for abdominal segmentation, and a modified U-Net architecture that was substituted with an attention mechanism [13] (OvGUMEMoRIAL) showed successful results for semantic segmentation of abdominal images.

## Methods

### Conditional GAN

In a conventional GAN, a generative model *G* learns a mapping from a random noise vector *z* to an output image *y*; $G: z \rightarrow y$. Meanwhile, a discriminative model *D* estimates the probability of a sample coming from the training data ($x_{real}$) rather than from the generator ($x_{fake}$). The objective function is a two-player mini-max game that can be formulated as

1 $$\begin{aligned}&\underset{G}{\min }\, \underset{D }{\max }\, V(G, D) = E_{x \sim p(\mathrm{data})} [\log D(x)]\nonumber \\&\qquad + E_{z \sim p(z)} [\log (1-D(G(z)))] \end{aligned}$$

In a conditional GAN (cGAN), a generative model learns a mapping from the random noise vector *z* and an observed image *x* to an output image *y*; $G: {x,z} \rightarrow y$. The discriminative model attempts to discriminate between the ground truth of the training set and the generator output as in a conventional GAN. The objective function conditions both *G* and *D* on the desired output *y*:

2 $$\begin{aligned}&\mathcal {L}_{adv} \leftarrow \underset{G}{m}in \, \underset{D }{m}ax \, V(G, D) \nonumber \\&\quad = E_{x,y \sim p_{data}(x,y)} [\log D(x,y)] \nonumber \\&\qquad + E_{z \sim p(z), y \sim p(y)} [\log (1-D(G(z,y),y))] \end{aligned}$$

In both conventional and conditional GAN frameworks, the task of a discriminator is much harder than that of the generator as it has to minimize the mistakes of the generator. Along with the mini-max nature of the objective, this raises several challenges such as mode collapse, vanishing gradient, and failure to converge. In this work, we propose a new framework to address the learning of an unbiased model on the class imbalance problem.

### Ensemble-GAN

**Fig. 1:**
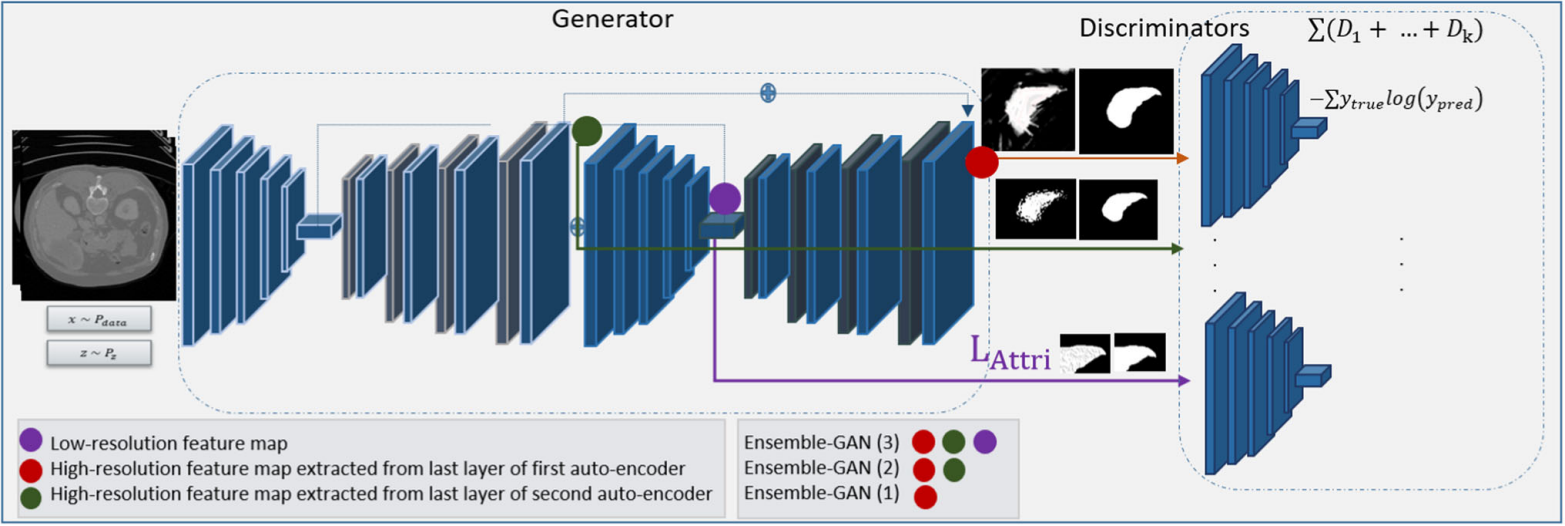
Overview of the architecture of the proposed Ensemble-GAN composed of a generator and multi-discriminator. The generator network (*G*) is a modified stacked hourglass architecture which takes random noise and medical images as the condition and tries to predict the semantic segmentation through an ensemble of *D* losses. Each *D* (with different losses) distinguishes between ground-truth and different global and local features map predicted by *G*


Figure 1 illustrates the architecture of our Ensemble-GAN where all components are parameterized by neural networks. The proposed framework consists of single-generator and multi-discriminator variants that attempt to better approximate $\max V(G, D_k)$, providing a better critic to the generator. Here, the generator learns from the feedback, aggregated over multiple discriminators either by $\sum _{k=1}^{K} V(G, D_k)$. The main idea of combining multiple discriminators in redundant ensembles is (1) to improve the generalization ability since each discriminator covers only some parts of the application data, (2) to combine multiple discriminators into a single consensus model (as maximum, average, or sum), which performs better than a single discriminator because the patterns that are misclassified by different discriminators are not the same, and (3) to overcome typical defects of vanilla conditional GANs, such as global structure collapse and local detail ambiguity by designing a new architecture for the generator.

In our workflow, the generator *G* is forced to learn to minimize the prediction error of semantic segmentation through the ensemble of discriminators. This ultimately encourages *G* to produce conditional samples with minimum error, since *G* needs to fool the different possible discriminators. Variations in the ensemble are achieved by the summation feedback of each *D* with a certain probability at the end of every batch. Therefore, *G* considers the sum of discriminator losses in the ensemble while updating its parameters at each iteration.

Similar to Luo et al. [15], the extracted local and global output by a single generator is passed into two individual discriminators. We designed and implemented different architectures with various losses based on our study. Increasing the number of discriminators (1) with different losses covers more aspects of the generator’s output by approximating $\sum _{k=1}^{K} V(G, D_k)$, and (2) with different representations of the data, they are also capable of better catching the distributions of the generator.

### The objective function

We formulate the proposed Ensemble-GAN with a cohort of three networks (see Fig. 1). Extension of the framework with more networks is discussed in “Experiments” section. Here, a single generator attempts to minimize the segmentation error regarding an ensemble of *k* different losses. The generator takes a random vector *z* and medical images *x* as input, whereas the discriminators attempt to minimize the error of predicting the segmentation masks produced by the generator through multiple losses. For a fixed *G*, function *F* will receive sum of *k* different discriminator losses to the generator through the objective of $\min _G \max _{D_k} F(V(D_1, G), V(D_2, G), \ldots , V (D_k, G)))$.

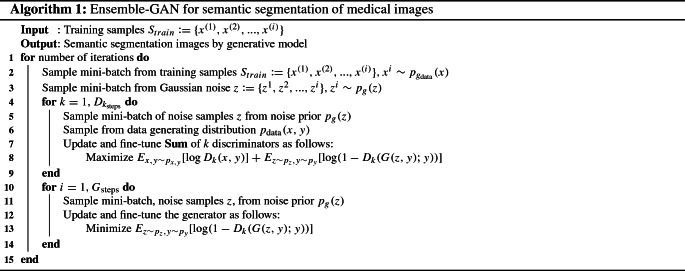

3 $$\begin{aligned}&\underset{G}{\min }\, \underset{D_k}{\max }\, V(D_k, G) = E_{x,y \sim p(x,y)} [\log D_k(x,y)] \nonumber \\&\qquad + \lambda _{k} E_{z \sim p(z) , y \sim p(y)} [\log (1-D_k(G(z,y),y))] \end{aligned}$$

The use of the proposed combination scheme prioritizes the worst discriminators and thus provides more useful gradients to the generator during the training. Details about the architectural choices, discriminator and generator losses, and the selection of the hyperparameter $\lambda$ are discussed in “Experiments” section.

## Experimental design

### Materials

We validated the performance of our proposed Ensemble-GAN based on clinical patient data from two recent, publicly available challenge datasets in abdominal imaging: (1) the automated liver and tumor segmentation (LiTS)^1^ of MICCAI 2017 conference and (2) the segmentation challenge (CHAOS)^2^ of the ISBI 2019 conference. Both datasets consist of abdominal CT and MR images for which each image slice has been manually segmented by expert radiologists.

**Fig. 2:**
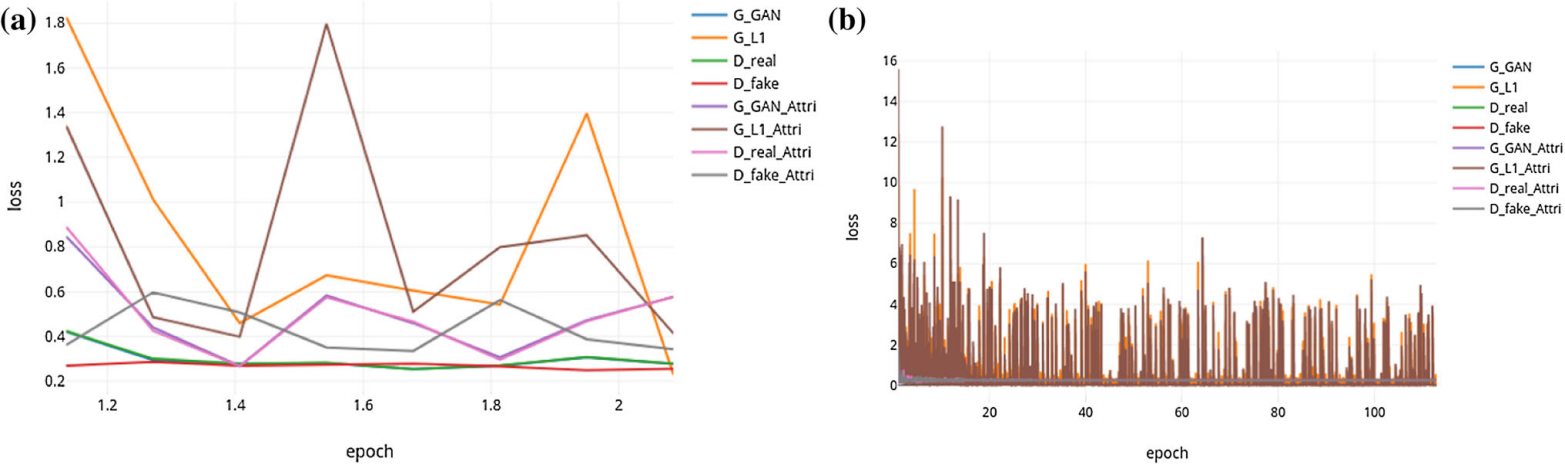
**a** In the earlier epochs of training of the Ensemble-GAN, when *G* improves, *D*s deteriorate because *G* and *D*s work against each other. **b** After several epochs of training, the ensemble of *D* reaches the point to improve segmentation output from *G*. As a result, the Ensemble-GAN shows a good convergence where the ensemble of *D*s is unable to differentiate between the real and fake distributions. Here, loss *G* indicates the loss of generator and loss $D_{real}$ and loss $D_{fake}$ indicate the adversarial losses of discriminator on real and fake image calculated on high-resolution features map, respectively. The *Attri* term denotes the losses calculated on low-resolution label map

#### CHAOS

The CHAOS challenge [16] is a Combined (CT-MR) Healthy Abdominal Organ Segmentation problem that has been organized into different segmentation tasks. In this study, we evaluated our model on the segmentation of abdominal organs (CT + MRI as a task (4)). The dataset included 20 MR and 20 CT abdominal images with five segmentation labels for the liver, spleen, left kidney, right kidney, and background. We trained our model on a total of 16,266 2D images with 256$\times$ 256 pixels and tested on 1,793 similarly sized 2D images. Here, the imbalanced ratios are 1:40, 1:200, 1:400, 1:400 defined as the number of pixels in the background class to the number of pixels belonging to the liver, spleen, left and right kidney.

#### LiTS

In the second experiment, we employed the LiTS-2017 dataset that contains 130 training and 70 test CT cases, in which patients were suffered from different types of liver cancers. The challenge was to perform a simultaneous semantic segmentation of a large liver that had a 1:400 imbalanced class ratio of pixels representing the liver and surrounding tissue with an abnormal target region with 1:1400 imbalanced class ratio between pixels representing abnormal and normal tissue.

### Experiments

We evaluated three architectural choices for the proposed Ensemble-GAN. The first experiment, Ensemble-GAN (1), included a single generator and two discriminators. As shown in Fig. 1, the generator had a stacked hourglass network design [17] which provides a mechanism for repeated bottom-up and top-down inference, allowing for a re-evaluation of the initial estimates and features across the whole image. The architecture of the discriminator was similar to a Markovian discriminator [18] to restrict the attention to the structure in local image patches. The discriminator losses were $\ell _{mae}$ and $\ell _\mathrm{Dice}$. For the hyperparameters, we set $\lambda _1 =10$ and $\lambda _2 =5$ for $D_1$ and $D_2$, respectively. We used a network pretrained with ImageNet for the initialization of the weights of the discriminators, but we trained the generator from scratch using a Gaussian distribution with a standard deviation of 0.001. The learning rate started from 0.0002 with a mini-batch size of 1. We used Adam [19] as the optimizer and set $\beta _1 = 0.9$, $\beta _2 = 0.999$ with a weight decay of 0.0001. We used the binary cross-entropy as the adversarial loss in all experiments.

The second experiment, Ensemble-GAN (2), included a single generator and three discriminators. The generator and discriminator networks had the same architecture as those of Ensemble-GAN (1). Here, we explored the effect of three discriminator losses on the outcome of the generator. We combined and added a categorical cross-entropy loss $\ell _{cce}$ as a third loss with $\lambda _3 = 25$. In this architecture, categorical cross-entropy calculates differentiation between the high-resolution feature map by last layer of first auto-encoder network and ground-truth images.

**Table 1:** Accuracy for simultaneous liver and lesions segmentation in terms of the Dice score and average surface distance on the test data, where 1 is the index for liver and 2 for lesions

Approaches	Dice1	Dice2	ASSD1	ASSD2
Ensemble-GANs (1)	0.91	0.80	1.4	1.9
Ensemble-GANs (2)	0.92	0.81	1.4	1.7
Ensemble-GANs (3)	0.94	0.84	1.3	1.6
cGAN	0.85	0.81	1.8	2.1
UNet	0.72	0.70	19.04	19.04
Cascaded-UNet [23]	0.93	0.93	2.3	2.3
UNet+3DCRF [23]	0.95	0.50	0.92	1.3
ResNet+Fusion [21]	0.95	0.50	0.84	13.33
SuperAI	0.96	0.81	–	1.1
H-Dense+ UNet [20]	0.96	0.82	1.45	1.1
coupleFCN [22]	0.78	0.77	–	–

**Table 2:** The top four rows show the accuracy of the liver segmentation

Architecture	VOE	RVD	ASSD	MSSD	F1	Precision	Recall	kappa
Ensemble-GANs (1)	17	$-$ 8	9.2	46.8	0.90	0.91	0.86	0.77
Ensemble-GANs (2)	16	$-$ 8	7.7	41.2	0.91	0.91	0.86	0.79
Ensemble-GANs (3)	14	$-$ 6	6.2	40.3	0.95	0.94	0.89	0.80
cGAN	21	$-$ 1	10.8	87.1	0.88	0.90	0.79	0.68
ResNet+Fusion [21]	16	$-$ 6	5.3	48.3	–	–	-	-
SuperAI	36	4.27	1.1	6.2	–	–	–	–
H-Dense+ UNet [20]	39	7.8	1.1	7.0	–	–	–	–
coupleFCN [22]	35	12	1.0	7.0	–	–	–	–

*VOE* volume overlap error, *RVD* relative volume difference , *ASSD* average symmetric surface distance, *MSSD* maximum symmetric surface distance

In the third experiment, Ensemble-GAN (3), three different outputs of a single generator were passed to three different discriminator losses. The generator and discriminator networks had the same architecture as those of Ensemble-GAN (1). We passed the second bottleneck and the last fully convolutional layer of each auto-encoder from generator separately as the output of the deep feature tensor and transferred them with the label map into three different discriminators. The feature vector of the bottleneck represents the local information of images, whereas the last fully convolutional layer contains global features. The combination of additional losses and the adversarial loss is controlled by a $\lambda$ hyperparameter, which controls the relative importance of each loss. Here, we used two categorical cross-entropy losses for the two different generator outputs: $\lambda _1 = 100$, $\lambda _2 = 25$ and $\lambda _2 = 1$ for high-resolution $\ell _{cce}$, low-resolution $\ell _{cce}$, and adversarial loss $\ell _{adv}$, respectively. Table 5 represents the effectiveness of $\lambda$ in semantic segmentation in terms of F1 score.

Figure 2 shows the training losses at the beginning and after 100 epochs.

**Fig. 3:**
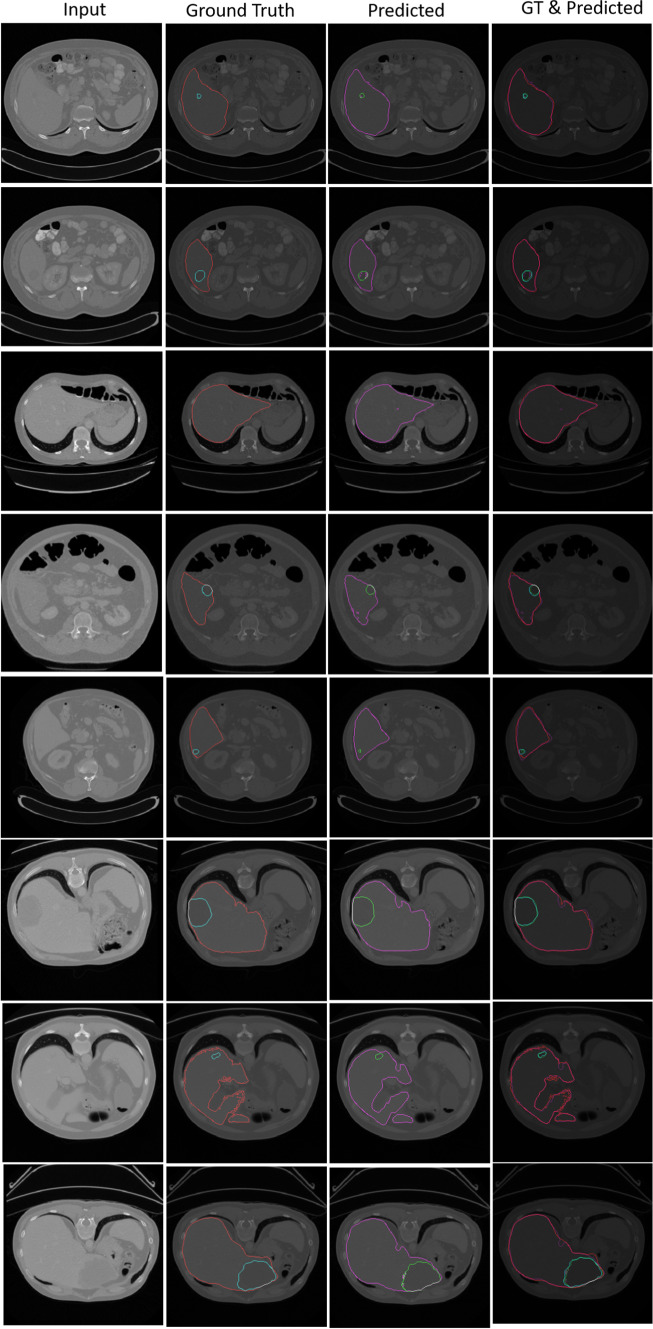
Semantic segmentation results obtained by Ensemble-GAN (3) on LiTS dataset

**Table 3:** The top three rows show the average accuracy for the semantic segmentation of abdominal CT and MR images with respect to the measurements obtained by the challenge organizer

Architecture	VOE	RAVD	ASSD	MSSD	DICE	F1	Precision	Recall
Ensemble-GAN (1)	0.14	6.9	6.1	39.1	0.91	0.95	0.96	0.89
Ensemble-GAN (2)	0.15	5.7	5.8	40.1	0.92	0.94	0.96	0.88
Ensemble-GAN (3)	0.12	3.1	2.9	32.1	0.94	0.96	0.97	0.90
cGAN	2	$-$ 1	10.8	17.3	51	0.83	0.85	0.69
PKDIA [13]	–	8.43	6.37	33.1	0.88	–	–	–
OvGUMEMoRIAL [13]	–	50	5.2	74.0	0.85	–	–	–
IITKGP-KLIV [13]	–	13.5	16.6	130	0.63	–	–	–
ISDUE [13]	–	14.0	9.81	37.1	0.85	–	–	–

*VOE* volume overlap error, *RAVD* relative volume absolute difference, *ASSD* average symmetric surface distance, *MSSD* maximum symmetric surface distance are defined by CHAOS organizers. The average F1 score, precision, and recall are calculated as measures for the handling of the class imbalance problem

**Table 4:** Effectiveness of each component and network architecture

Architecture	CHAOS				LiTS	
	Liver	Spleen	Right kidney	Left kidney	Liver	Lesion
Conditional GAN						
1 Disc. $L_{mae}$	0.88 ± 0.08	0.80 ± 0.08	0.84 ± 0.09	0.91 ± 0.02	0.87 ± 0.02	0.82 ± 0.11
1 Disc. $L_{Dice}$	0.89 ± 0.06	0.83 ± 0.12	0.86 ± 0.03	0.92 ± 0.05	0.88 ± 0.01	0.84 ± 0.05
1 Disc. $L_{cce}$	0.89 ± 0.03	0.81 ± 0.14	0.85 ± 0.08	0.92 ± 0.03	0.88 ± 0.02	0.83 ± 0.07
1 Disc. $L_{bce}$	0.87 ± 0.14	0.77 ± 0.20	0.83 ± 0.05	0.90 ± 0.05	0.86 ± 0.02	0.82 ± 0.04
Cyclic-Ensemble-GAN						
2 Disc. $$ L_{mae}+ L_{adv}$$	0.89 ± 0.05	0.88 ± 0.03	0.91 ± 0.06	0.91 ± 0.04	0.89 ± 0.01	0.85 ± 0.08
Ensemble-GAN (1)						
2 Disc. $L_{mae} + L_{Dice}$	0.89 ± 0.02	0.87 ± 0.10	0.90 ± 0.04	0.91 ± 0.09	0.92 ± 0.07	0.84 ± 0.02
2 Disc. $L_{bce} + L_{Dice}$	0.89 ± 0.04	0.88 ± 0.06	0.91 ± 0.03	0.92 ± 0.03	0.93 ± 0.01	0.86 ± 0.23
2 Disc.$L_{1Attri} + L_{cce}$	0.92 ± 0.02	0.89 ± 0.05	0.92 ± 0.02	**0.94** ± **0.02**	0.93 ± 0.02	0.85 ± 0.22
2 Disc. $L_{1Attri}+ L_{bce}$	0.91 ± 0.03	0.88 ± 0.02	0.91 ± 0.14	0.93 ± 0.03	0.93 ± 0.02	0.85 ± 0.05
Ensemble-GAN (2)						
3 Disc. $L_1 + L_{cce} + L_{Dice}$	0.92 ± 0.02	0.90 ± 0.12	0.91 ± 0.05	0.94 ± 0.03	0.92 ± 0.02	0.88 ± 0.02
Ensemble-GAN (3)						
3 Disc. $L_1 + L_{cce}+ L_{1Attri}$	**0.95** ± **0.05**	0.92 ± 0.03	**0.93** ± **0.02**	0.94 ± 0.04	0.96 ± 0.07	0.89 ± 0.02
3 Disc. $L_1 + L_{Focal}+ L_{1Attri}$	0.94 ± 0.08	**0.93** ± **0.03**	0.93 ± 0.06	0.93 ± 0.05	**0.96** ± **0.02**	**0.90** ± **0.01**

The F1 scores obtained across 100 epochs using the different datasets with different imbalanced ratios and image modalities are shown in the table. Bold scores indicate the best F1 score obtained for each dataset

We implemented the Ensemble-GAN on top of Macro–Micro GANs [15]. We used all 2D slices from the axial view with size $256 \times 256$ for the CHAOS dataset and $512 \times 512$ for the LiTS dataset. For data augmentation, we applied random cropping, mirroring, scaling, enhancement, and $[-10,+10]$ degree random rotation in all the experiments. The networks were trained on a workstation equipped with five Nvidia Titan X GPUs.

### Evaluation criteria

The evaluation and comparison of the Ensemble-GAN were performed using the quality metrics introduced by each challenge organizer. We evaluated the performance of the proposed method with the F1 score and precision–recall as a measure for handling the imbalanced issue.

For the LiTS competition, the primary metric was the Dice score. A volume overlap error (VOE), relative volume difference (RVD), average symmetric surface distance (ASSD), and maximum symmetric surface distance (MSSD) were considered for the evaluation of the predicted region of the liver and the liver lesions. Tables 1 and 2 describe the quantitative results and comparisons with top-ranked methods from the LiTS leaderboard.^3^

Among the four metrics determined by the CHAOS organizer for evaluating the multi-organ segmentation,^4^ Dice coefficient, average symmetric surface distance (ASSD), relative volume difference (RVD), and maximum symmetric surface distance (MSSD) were utilized to determine the potential over- and under-segmentation boundaries.

## Results

To understand the performance gains, we analyzed the accuracy on the imbalanced liver tumor segmentation dataset, where we can see the unbalanced labels between the large organs and very small lesions. Based on the leaderboard, most of the top-ranked models used cascade networks to segment the liver and the lesions simultaneously [20] or separately [21, 22]. The generative ensemble networks provided a good solution against the imbalanced labeling.

Table 1 shows the Dice scores for the liver and lesion segmentation. The highest scores obtained by our proposed framework were 0.94 for the liver and 0.84 for the lesions. Based on a comparison of the first two rows of Table 1, we find that the effect of the ensemble of discriminators on the final result increased up to 9% for the liver segmentation and up to 3% for the segmentation of lesions.

For the LiTS dataset, lesions with an approximate diameter equal to or larger than 10 mm were defined as large, while lesions with a diameter of less than 10 mm were defined as small. Our method achieved an average Dice score of 0.91 and an ASSD of 1.4 in the lesion segmentation, indicating that the method can distinguish between small and large lesions.

The heterogeneous structures of the predicted liver and all lesions from the local test set are shown in Fig. 3. We used a fivefold cross-validation for the training due to the different intensity distributions of the cases. In the test phase, the voted average of these models was used for making a prediction for each case in the test dataset.

**Fig. 4:**
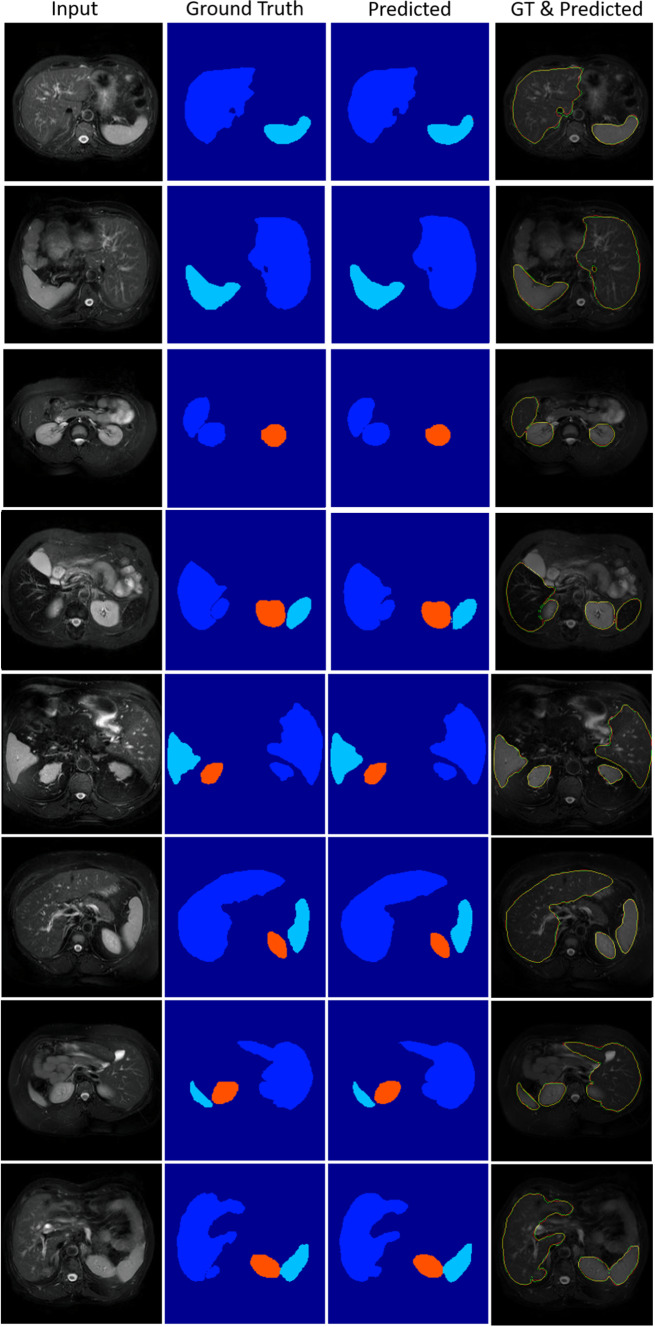
Semantic segmentation results obtained by Ensemble-GAN (3) on CHAOS dataset

**Fig. 5:**
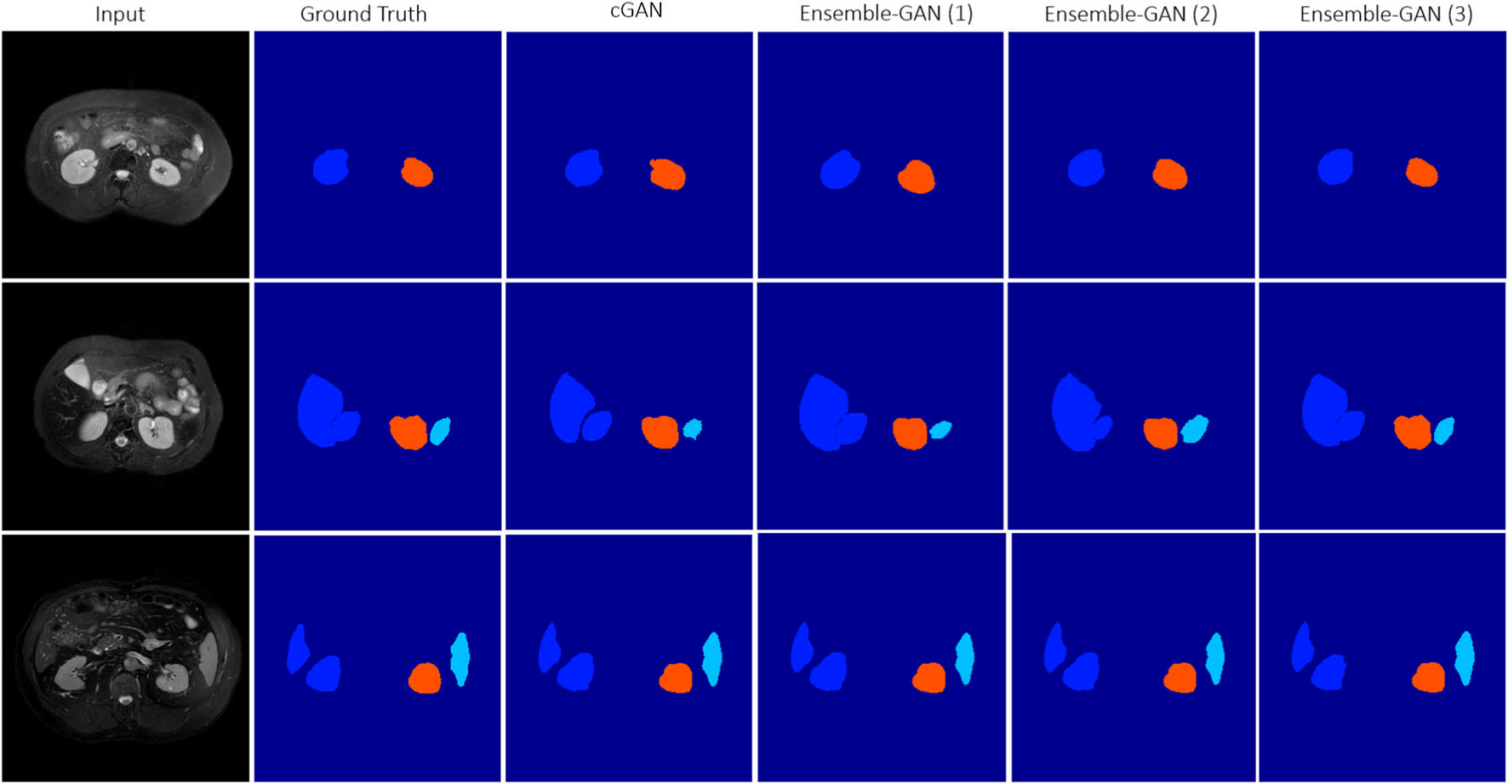
Different losses induce different qualities of results. Each column shows the results predicted by different models

**Table 5:** Effectiveness of hyperparameter $\lambda$ on semantic segmentation results in terms of F1 score

Architecture	CHAOS				LiTS	
	Liver	Spleen	r-kidney	l-kidney	Liver	Lesion
Conditional GAN						
1 Disc. $L_{bce}$	0.86	0.77	0.83	0.89	0.84	0.82
Ensemble-GAN (1)						
$$\lambda _1 = 1$$						
2 Disc. $\lambda _1 L_{mae} + L_{bce}$	0.89	0.88	0.90	0.91	0.90	0.84
$$\lambda _1 = 10$$						
2 Disc. $\lambda _1 L_{mae} + L_{bce}$	0.89	0.89	0.91	0.91	0.91	0.85
$$\lambda _1 = 100$$						
2 Disc. $\lambda _1 L_{mae} + L_{bce}$	0.90	0.89	0.91	0.92	0.91	0.85
Ensemble-GAN (3)						
$$\lambda _1 = 1, \lambda _2 = 1, \lambda _3 = 1$$						
3 Disc. $\lambda _1L_{mae} +\lambda _2 L_{cce}+ \lambda _3 L_{1Attri}$	0.94	0.90	0.93	0.94	0.95	0.87
$$\lambda _1 = 10, \lambda _2 = 10, \lambda _3 = 10$$						
3 Disc. $\lambda _1L_{mae} +\lambda _2 L_{cce}+ \lambda _3 L_{1Attri}$	0.95	0.91	0.93	0.94	0.96	0.89
$$\lambda _1 = 25, \lambda _2 = 25, \lambda _3 = 100$$						
3 Disc. $\lambda _1L_{mae} + \lambda _2 L_{cce}+\lambda _3 L_{1Attri}$	0.95	0.92	0.93	0.94	0.96	0.89

The F1 scores obtained across 100 epochs on both datasets are shown in the table

The top three rows of Table 3 show the quantitative results achieved by the different Ensemble-GAN architectures. According to Table 3 and Fig. 4, the predicted semantic segmentation by Ensemble-GAN (3) outperformed the other architectures, which demonstrates the success of passing the dual output of the generator as global and one output of local feature vectors into three individual pretrained discriminators. The local features include more details on edges, while the global features contain more localized features. Having two adversarial losses for global and local discriminators besides binary cross-entropy of generative model leads to a better recognition and smoother boundaries of segmentation in both benchmarks than with the other approaches. As reported by CHAOS [13], the three top-ranked teams in task 4 used different deep ensemble discriminator networks such as cascade architectures and therefore reported a more stable result in the test phase. The achieved Cohen’s kappa scores by Ensemble-GAN (1–3) on CHAOS dataset are 0.77, 0.78, and 0.8, respectively, where the average kappa score is 0.64 by the conditional GAN.

Figure 5 and Table 4 represent and compare qualitative and quantitative results achieved by different Ensemble-GAN setting and configuration.

The results showed (Table 5) that choosing larger $\lambda$ can generate more accurate semantic segmentation images. The adversarial loss influences if the generator model can output images that are acceptable in the target domain. Therefore, the combination of other losses and adversarial loss regularizes the generator model to output images that are an acceptable translation of the source image. We controlled the impact of additional losses by a $\lambda$ hyperparameter, where set to 10 means giving ten times importance of $L_{mae}$ loss than the adversarial loss during training and testing. To explore effect of hyperparameter of $\lambda$ in the task of medical image semantic segmentation, we did several experiments shown in Table 5 in terms of F1 score.

## Discussions and conclusions

In this study, we introduced a novel Ensemble-GAN framework to mitigate the issues introduced by an imbalanced training set. The Ensemble-GAN framework enables a single generator to learn from an ensemble of discriminators that differ by initialization, loss, and subsets of the training data. The Ensemble-GAN enhances the prior developments of the MD-GAN [12] and Micro–Macro GAN [15] by its different network architecture and the handling of imbalanced data.

Our experiments on multiple datasets demonstrated that the Ensemble-GAN greatly alleviates the imbalanced data problem and provides better generalization than existing approaches in the semantic segmentation of CT and MR images. Compared to a conditional GAN, the Ensemble-GAN also increases the stability of training over time by enabling the generator to receive more feedback from the discriminators.

Moreover, we introduced various modifications to conditional GAN that lead to better trade-off between precision and recall, thereby preventing local and global inconsistency in the output prediction. Our segmentation results on two popular abdominal benchmarks indicate that the Ensemble-GAN is robust with respect to global inconsistencies such as slice misalignment and different image protocols, as well as to local inconsistencies such as blurring of the images. Given its high accuracy, the Ensemble-GAN has the potential to be practically useful in clinical routine. In future work, we would like to investigate the prediction of semantic segmentation by ensemble generators that learn from an ensemble of discriminators through adversarial process. A study of the implications of using STAPLE [24] on top of a fixed generator that would receive an average of different discriminator losses would be another topic for a future study.

## Footnotes

1 https://chaos.grand-challenge.org/.

2 https://competitions.codalab.org/competitions/17094.

3 https://competitions.codalab.org/competitions/.

4 https://github.com/emrekavur/CHAOS-evaluation.
